# Oligocene moisture variations as evidenced by an aeolian dust sequence in Inner Mongolia, China

**DOI:** 10.1038/s41598-022-09362-y

**Published:** 2022-04-04

**Authors:** Joonas Wasiljeff, Johanna M. Salminen, Jarkko Stenman, Zhaoqun Zhang, Anu Kaakinen

**Affiliations:** 1 Department of Geosciences and Geography, University of Helsinki, P.O. Box 64, 00014 Helsinki, Finland; 2 Present Address: Geological Survey of Finland, P.O. Box 96, 02151 Espoo, Finland; 3 Present Address: Department of Civil Engineering, Aalto University, P.O. Box 11000, 00076 Aalto, Finland; 4 Key Laboratory of Vertebrate Evolution and Human Origin of the Chinese Academy of Sciences, Institute of Vertebrate Paleontology and Paleoanthropology, Chinese Academy of Sciences, Beijing, 100044 China; 5 CAS Center for Excellence in Life and Paleoenvironment, Beijing, China; 6 University of Chinese Academy of Sciences, Beijing, China

**Keywords:** Geology, Palaeomagnetism, Geochemistry

## Abstract

The aridification of Central Asia since the Eocene has widespread evidence, but climate-controlled environmental reorganizations during the Oligocene remain ambiguous. We employed environmental magnetic, mineralogical and geochemical methods on a latest Eocene to late Oligocene terrestrial sequence in Inner Mongolia, China, to examine how global climatic trends and regional factors influenced the evolution of moisture and weathering in the region. Highlighting the climatic influence, our weathering and rainfall proxy data document the drawdown of atmospheric CO_2_ and global cooling during the early Oligocene semi-arid phase, which culminated in the Early Oligocene Aridification Event at 31 Ma. Moreover, for the first time in the terrestrial eastern Central Asian setting, we provide geochemical and geophysical evidence for a second major Oligocene aridification event nearly synchronous to the mid-Oligocene Glacial Maximum at around 28 Ma. These aridification events were interrupted by periods of increased rainfall and weathering and can be associated with the terminations of glacial events seen in marine oxygen isotope records.

## Introduction

In the aftermath of the climatic and environmental reorganization at the Eocene–Oligocene Transition (EOT) ~ 34 Ma^1–3^, the Oligocene epoch was marked by dramatic shifts in the global climate, as indicated in the Southern Hemisphere by large-scale ice sheet oscillations^4,5^ and largely variable marine and terrestrial surface temperatures^6^. The epoch was characterized by strong, ~ 1.2 My obliquity and relatively weaker precession cycles, as expressed by the recurring glacial episodes, both beginning from the Oi-1 glacial ~ 33.8–33.6 Ma^5,7–9^. From then on, the Asian terrestrial climate during the Oligocene was possibly driven by glacial–interglacial ice-sheet advances and retreats over the precession and obliquity cycles in response to summer insolation, inducing variations in the sea level, atmospheric and oceanic circulation, CO_2_ and temperature^8^.

Nevertheless, our knowledge of the terrestrial systems of the Oligocene remains temporally limited and any environmental reconstructions are spatially sparse, which has led to substantial debate on the cause and extent of environmental impacts, such as the aridification of the Asian interior^3,10–16^. Since the Tibetan Plateau was likely to have been a generally rather low-lying but topographically highly varied landscape during the Eocene to late Oligocene^17–19^, its role in the drying climate of the Asian interior during the Palaeogene remains ambiguous. Instead, the development of aridity around the EOT has been explained by the combined effects of global cooling^3,9,16^ and stepwise retreat of the proto-Paratethys Sea^20^. The latter process was primarily driven by tectonism^21^, while the sea’s high and low stands were modulated by changes in the Antarctic ice sheets^3,9,16,22^. The retreat would have then decreased the available moisture^20^, which was only transported in low amounts by the prevailing Westerlies, hence amplifying the aridity^23,24^ and leading to the widespread loss of vegetation cover across the EOT, restricting it to higher elevations during the Oligocene in Central Asia^25^. The westward retreat of the sea has been considered to have increased the land–sea contrast, hence influencing the formation of the Asian monsoon^26^. However, the Oligocene climate of northwestern China and nearby regions has been considered humid and the terrains forest covered^19,27^. Hence, the importance of the proto-Paratethys Sea to the aridification process has been questioned^19^, as the retreating sea should have caused a permanent rather than a transient impact on aridification. This discrepancy could be resolved by recent evidence of a much larger Oligocene Paratethys Sea than previously understood^28^, but its role as a proximal Oligocene moisture source requires further elaboration.

Dust deposits are valuable archives of palaeoenvironmental information, including continental aridity, glacial conditions and dominant wind patterns^29^. These deposits are copious in the Eocene–Oligocene of Central Asia, making it an important region to study terrestrial deep time environments^13,15,30,31^. One of these archives, the Ulantatal area in Alashan, Inner Mongolia, China, provides an opportunity to investigate long-span environmental changes in a continuous sequence of windblown fine-grained deposits hosting numerous fossil localities in stratigraphic superposition^15,32^. The time span of the sediments of Ulantatal extends from the latest Eocene to the late Oligocene, ca. 35 to 27 Ma^15^. The current climate in the Alashan area is typical for temperate desert-steppe regions, and it has mean annual precipitation (MAP) of 210.2 ± 56.7 mm in the southwest and 35.2 ± 19.5 mm in the northwest near the Gobi desert^33^. The area may already have been part of the west–east-trending Central Asian arid belt during the late Eocene and early Oligocene^15,34^.

In this paper, we utilize a combined environmental magnetic, mineralogical and geochemical approach and present evidence for climate-controlled phases of increased and decreased aridity in the Ulantatal region. Iron oxides, including magnetite, maghemite, hematite and goethite, are a well-known tool to reconstruct variations in palaeorainfall and the palaeoclimate from aeolian dust deposits^35–39^. In addition, the concentration of organic matter in the aeolian deposits of arid and semiarid regions is suggested to reflect the vegetation cover history and biomass variations^40,41^, as well as effective moisture^42^. To further our interpretations of changes in the palaeoclimate, we utilized the geochemical MAP model RF-MAP2.0^43^_,_ based on the bulk sediment major element composition, to estimate past rainfall in the Ulantatal region. In addition, K-feldspar, plagioclase and quartz abundances were determined to capture changes in weathering intensity. Our multiproxy results show how the regional terrestrial environment responded to Oligocene climate dynamics, and we investigate the potential role of the proto-Paratethys Sea as a moisture source during the late Oligocene.

### Geological setting

**Figure 1:**
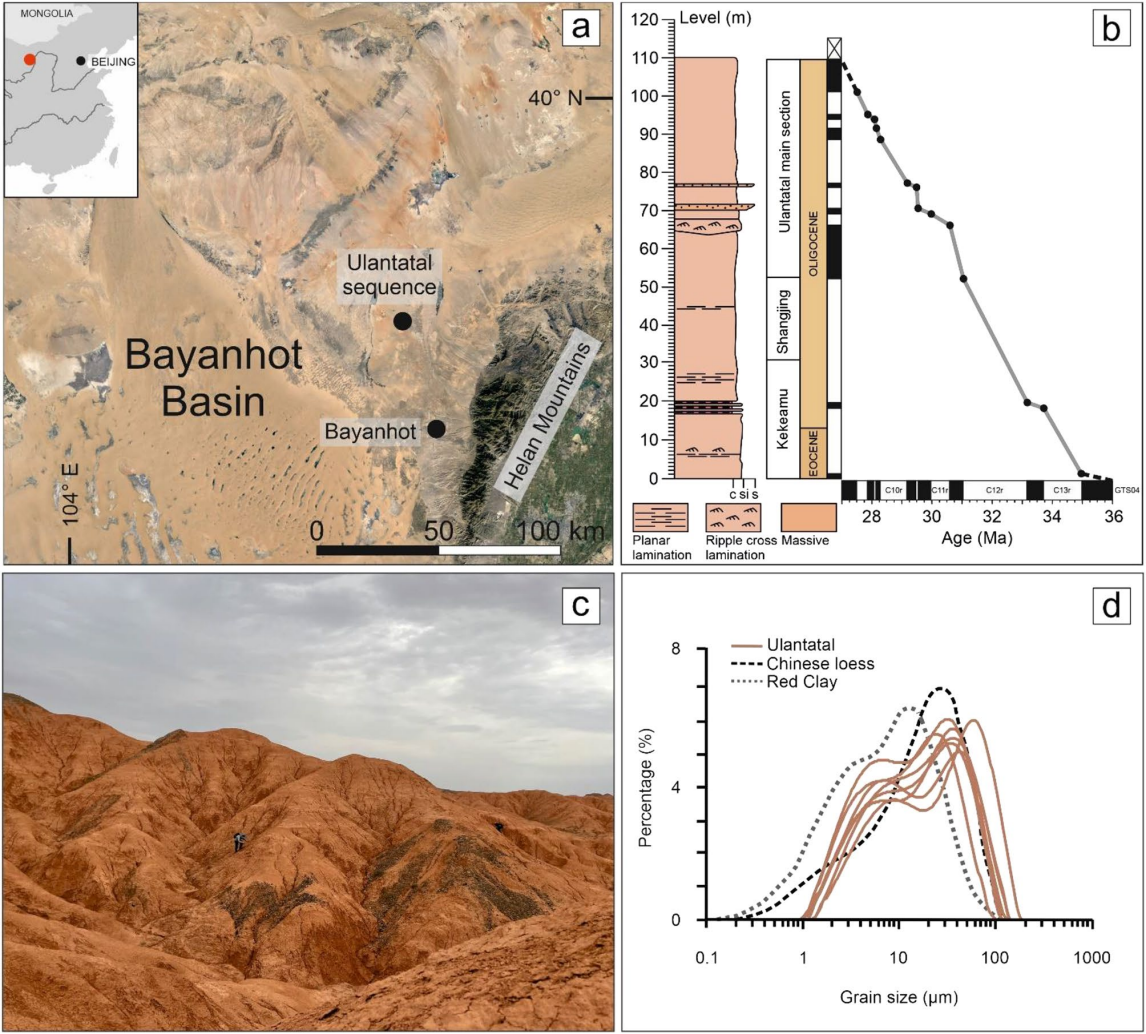
(**a**) A regional Google Earth Pro map (www.google.com/intl/en/earth) of the Bayanhot Basin, also showing the location of the studied Ulantatal sequence. The satellite image was modified with CorelDRAW 2020 (www.coreldraw.com) (**b**) A simplified lithological column of the Ulantatal sequence with subsections, sediment thickness, epochs and age-depth model. **c** Field view from the Kekeamu section where alternating more yellowish versus more reddish beds are visible. (**d**) Grain size distributions of selected samples from Ulantatal in comparison to Chinese loess and Red Clay. (**b**, **d**) are reproduced from^15^.

The studied Ulantatal sequence is situated in Alashan, in the north-central part of China, in the autonomous region of Inner Mongolia, sitting between the Tibetan Plateau and the Chinese Loess Plateau (Fig. 1a). The area resides in the eastern parts of the Bayanhot Basin of the Alxa Block, next to the north–northeast-trending Helan Mountains^15^. The Helan Mountains separate the basin from the Ordos and Yinchuan Basins in the east, and the Bayanwulan Mountain from the Yin’E and Chaoshui Basins in the northwest, while the Cha-Gu fault lies in the south.

The basin has experienced four major stages of evolution, starting from the aulacogen stage in the early Palaeozoic, followed by a marine to continental depression basin stage in the late Palaeozoic, a rift-depression basin in the Mesozoic, and finally to the current depression basin in the Cenozoic^44^. Even though Bayanhot Basin experienced multiple-staged tectonic movements during the Phanerozoic^44^, the time interval between the latest Eocene to late Oligocene was relatively stagnant allowing the extensive deposition of aeolian dust^15^, apart from the minor uplift event of the Helan Mountains around 30 Ma^45^. Based on fission-track dating of Zongbieli-Zhenggyiguan Fault of the Helan Mountains, a major uplift of the mountains occurred likely during the Miocene^46^.

A thick Cenozoic sedimentary cover dominantly overlies the region near the Ulantatal sequence, and its landscape can be best described as open with little topographic relief, outlined by a series of low gullies. The Ulantatal sequence unconformably overlies the Eocene Qaganbulag Formation, with a uniform and gentle dip angle towards the southwest. As revealed by our previous bio- and magnetostratigraphic study, the age span in the sediments is ca. 35–27 Ma^15^, inferred from an age–depth model established with piecewise linear interpolation (Fig. 1b). Ulantatal is composed of three sections: Kekeamu, Shangjing and the Ulantatal main section (Fig. 1b). The lowest part of the sequence is composed of coarse silts associated with decimetre-scale sand intercalations, which are considered to represent floodplain deposition. These fluvial deposits show a gradational upsection change into laterally and vertically continuous and texturally uniform, structureless silts, which are interpreted to mainly originate from aeolian dust deposition^15^. Grain size and textural characteristics show close similarity with Neogene Red Clays and Quaternary loess–palaeosol sequences^15^ with grain-size modes and bimodal distributions similar to typical loess deposits (Fig. 1d). In general, these deposits have subtly alternating colours (Fig. 1c), from reddish yellow (Munsell colours 5YR 6/6 and 5/6) to reddish (Munsell colours 2.5YR 5/6 and 6/6), with the latter displaying more pedogenic alteration, redoximorphic features and signs of burrows, while the yellowish sediments are more calcareous. Furthermore, the deposits are fossiliferous, with profuse small mammal fossils across all of the sequence, in addition to the sporadic presence of larger mammals^15,32,47^.

The lowest part of the Kekeamu section displays floodplain characteristics, consisting of coarse silt interfingering with sub-metre-thick, fine-grained sand units and occasional thin parallel lamination and ripple-cross lamination. However, in most of the section, especially above the Eocene–Oligocene Boundary, the well-cemented siltstones are mostly massive and often slightly calcareous. Occasional marks of burrowing, convolute laminations and rip-up clasts occur in the upper part of the Kekeamu section. The Shangjing section, on the other hand, is highly homogeneous, consisting of massive yellowish-brown-reddish siltstones with some slightly calcareous beds in the lower part of the section. The Ulantatal main section is composed of massive variegated clayey silt and silt beds with occasional intercalations of thin parallel and cross-laminated fine sand lenses. A thick erosional feature with a sharp contact, comprised of well-sorted coarse silt, overlays the thick siltstones in the lower-middle part of the Ulantatal main section. Above this coarser bed, a distinctive 3-m-thick dark red (Munsell colour 2.5YR 3/6) clayey silt unit, dated to ca. 29–30 Ma, is prevalent across several kilometres along the outcrops. This bed also displays abundant features of pedogenesis, such as slickensides and peds, as well as rip-up clasts. The topmost part of the Ulantatal main section is dominantly massive, sporadically calcareous, and shows subtle alternating reddish-yellow and reddish colours.

For this study, we collected sediment samples across the whole sequence for geochemical, mineralogical and mineral magnetic measurements from fresh exposed surfaces dug at least 50 cm into the outcrop.

## Results and interpretations

### Magnetic properties

**Figure 2:**
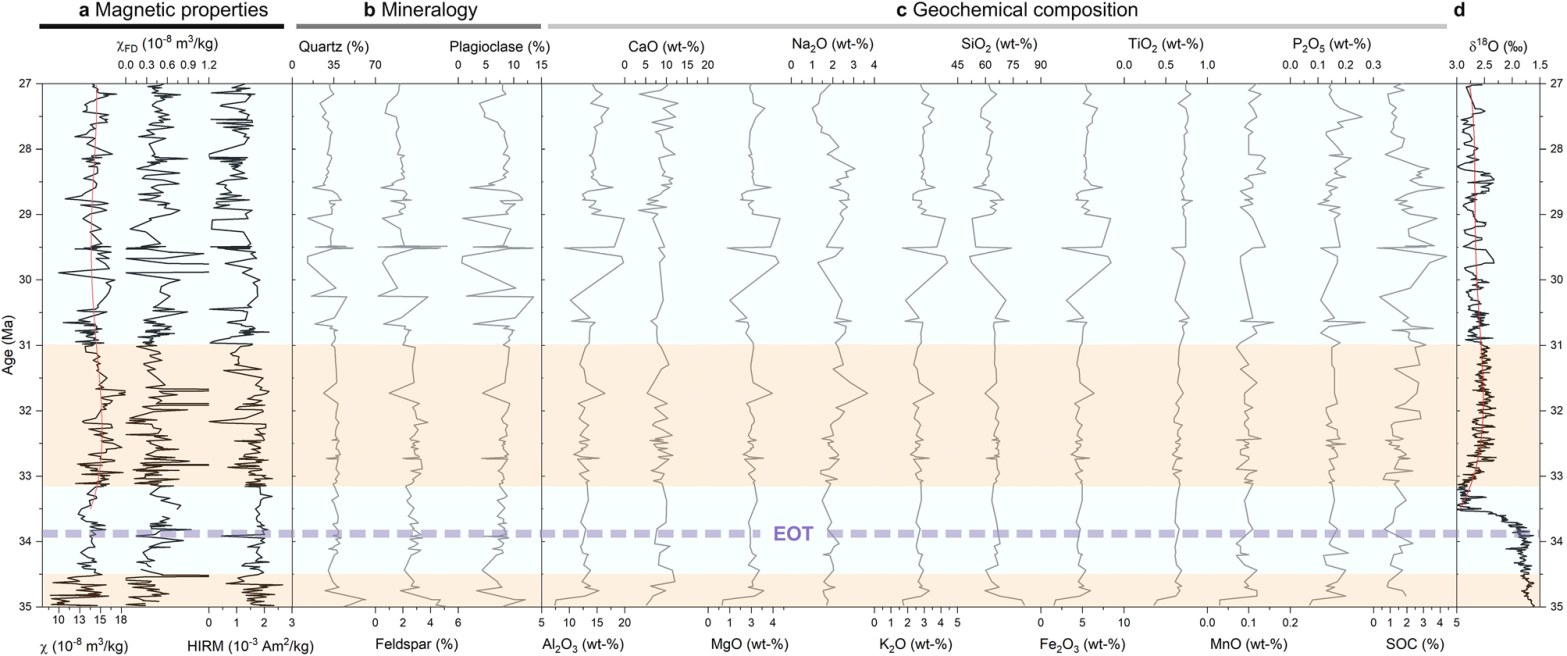
Palaeoenvironmental records during the latest Eocene to late Oligocene. (**a**) Magnetic records showing mass-normalized low-frequency magnetic susceptibility (χ), the absolute frequency dependency of magnetic susceptibility (χ_FD_) and hard isothermal remanent magnetization (HIRM). (**b**) K-feldspar, plagioclase and quartz abundance. (**c**) Major elemental oxides (Al_2_O_3_, CaO, MgO, Na_2_O, K_2_O, SiO_2_, Fe_2_O_3_, TiO_2_, MnO, P_2_O_5_) and the soil organic carbon (SOC) content. (**d**) The compiled benthic foraminifer δ^18^O record^2^. The red line in (**a**, **d**) from 33.5 to 27 Ma is the fourth-order polynomial fit to show the trend. Four zones based on the proxies are marked on the figure.

Based on the stratigraphic variation in bulk magnetic properties, four distinct zones can be defined (Fig. 2a). The basal part of the sequence (35–34.5 Ma) is characterized by the lowest mean mass normalized low-field magnetic susceptibility (χ) values obtained for the succession, with a relatively large amplitude of difference (mean: 12.5 × 10^−8^ m^3^/kg, avedev: 0.18 × 10^−8^ m^3^/kg). Slightly higher values of χ with less variation are obtained across the EOT at 34.5–33.1 Ma (mean: 13.7 × 10^−8^ m^3^/kg, avedev: 0.06 × 10^−8^ m^3^/kg). The following interval (33.1–31.0 Ma) marks the highest χ values and greater variation (mean: 15.1 × 10^−8^ m^3^/kg, avedev: 0.09 × 10^−8^ m^3^/kg), while slightly lower values are observed for the upper half of the sequence (31.0–26.9 Ma, mean: 14.1 × 10^−8^ m^3^/kg, avedev: 0.08 × 10^−8^ m^3^/kg). χ is dependent on the concentration of all magnetic minerals, but as ferrimagnetic minerals have a higher intrinsic magnetization than antiferromagnetic minerals, χ mostly reflects changes in ferrimagnetic minerals, e.g.^48^. Moreover, since χ is induced magnetization (measured in the presence of a magnetic field), it includes contributions from magnetic minerals of all grain sizes.

Wet and dry cycling in well-drained soils promotes bacterially induced redox processes that may produce nanoscale (superparamagnetic, SP) magnetite^49–52^. The presence of these grains can be detected with frequency-dependent magnetic susceptibility (χ_FD_) and with first-order reversal curve (FORC) analyses (Supplementary Fig. S1). SP ferrimagnets are often associated with a pedogenic origin^35–37^. These pedogenic ferrimagnetic magnetites are integrated into the pre-existing population of coarser-grained detrital magnetic minerals, e.g.^51^. In the Ulantatal sequence, the amount of nanoscale magnetite is moderate throughout the sequence, as evidenced by the relative χ_FD_ (mean: 3.0%; avedev: 0.9%) (Supplementary Fig. S2), and the absolute χ_FD_ largely mirrors the fluctuation in χ values, implying that magnetic susceptibility is partly governed by the nanoscale magnetite (Fig. 2a).

The magnetic mineral composition was examined by analysing hard isothermal remanent magnetization (HIRM) (Fig. 2a), the L-ratio and S-ratio^37^ (Supplementary Fig. S2) and thermomagnetic curves (Supplementary Fig. S4). HIRM reflects the contribution of high coercivity antiferromagnetic remanence carriers, commonly hematite and goethite, whilst the L-ratio records the coercivity changes of these minerals. If HIRM does not correlate with the L-ratio, HIRM can be considered as a proxy for hematite abundance^53^. The S-ratio, in turn, is a measure of the relative amount of ferrimagnetic (low-coercivity; e.g., magnetite and maghemite) and antiferromagnetic (high-coercivity; e.g., goethite and hematite) minerals in the total magnetic mineral assemblage^54^. When the S-ratio is close to 0.5 (close to zero), the contribution of ferrimagnetic (antiferromagnetic) minerals dominates^53^. In Ulantatal, the L-ratio is constant (mean 0.78) from 34.5 Ma onwards, including the EOT, and the L-ratio and HIRM are uncorrelated (Supplementary Fig. S4). Thus, HIRM reflects the amount of hematite. The S-ratio is equally stable (mean 0.3) and indicates that ferrimagnetic minerals dominate the magnetic properties of the sediments throughout the sequence. While χ and χ_FD_ exhibit a similar pattern of temporal variation and higher values after the EOT, HIRM displays lower values in the upper part during ca. 30–27 Ma. This decoupled behaviour between HIRM and magnetic susceptibility (χ and χ_FD_) implies a lower hematite abundance and higher ferrimagnetic mineral (magnetite/maghemite) proportions during the late Oligocene. The susceptibility versus temperature (χ-T) experiments on selected samples further support the presence of ferrimagnetic magnetite and antiferromagnetic hematite (Supplementary Fig. S4) throughout the sequence. All the samples show a loss of susceptibility at 560–580 °C, near the Curie temperature of magnetite, and at ca. 640–680 °C, indicative of the Néel temperature of hematite^55^. Occasionally, an increase in susceptibility before a peak at around 300 °C is observed, which can result from gradual unblocking of nanoscale ferrimagnetic grains^56^. Goethite is present in some samples, as indicated by the decrease in susceptibility between ca. 70 and 130 °C^57^.

### Palaeorainfall

**Figure 3:**
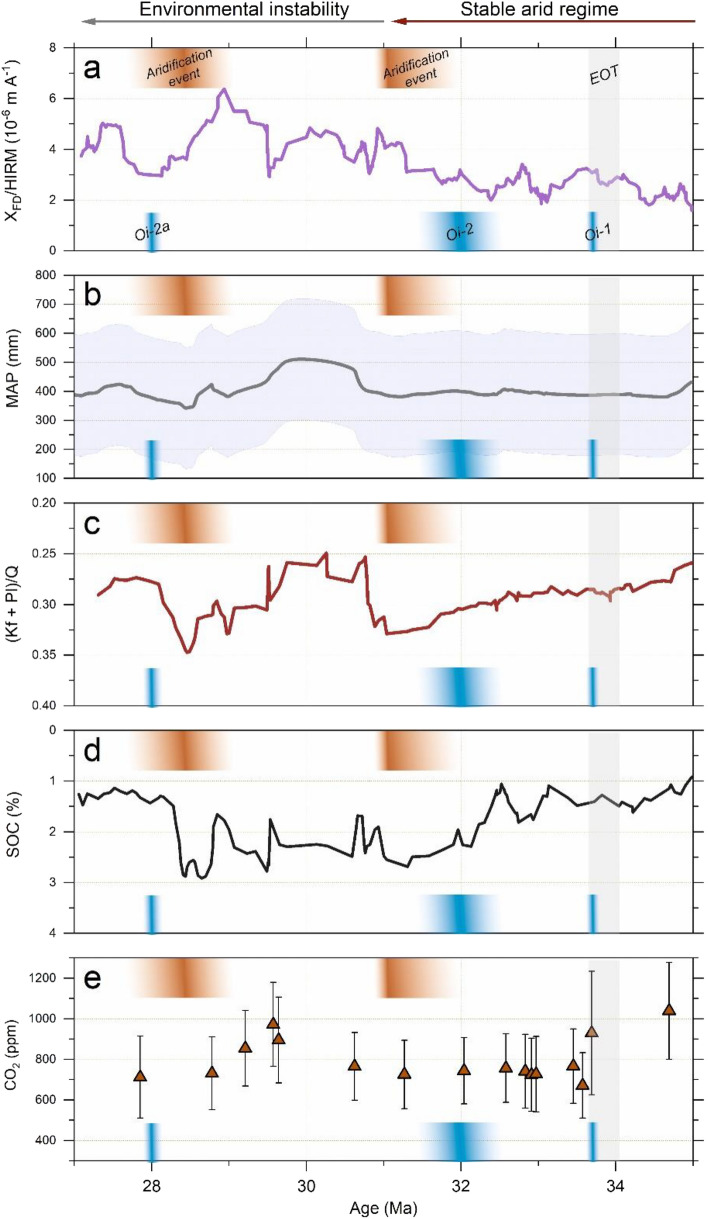
Changes in aridity within the Bayanhot Basin inferred from (**a**) the χ_FD_/HIRM ratio. The curve shown is constructed from data filtered from outliers and smoothed with a 10-point running average. (**b**) Mean annual precipitation (MAP) based on the RF-MAP2.0 model^43^, with the shaded area indicating an error of ± 209 mm. (**c**) Weathering intensity as indicated by the ratio of feldspars to quartz (Kf + Pl)/Q. (**d**) The soil organic carbon (SOC) content. In (**b**–**d**) the curve is smoothed with a 5-point running average. (**e**) Atmospheric CO_2_ reconstruction based on alkenones (orange triangles)^59^. The EOT and Oi-1^8^, Oi-2 and Oi-2a glaciations^60–62^, as well as the two major aridification events are indicated in the figure. Note that the y-axis scale in panels (**c**, **d**) is reversed.

Rainfall characteristics were investigated by calculating the χ_FD_/HIRM ratio and using the geochemical RF-MAP2.0 model^43^. The ratio of SP ferrimagnets and hematite can be expressed with χ_FD_/HIRM, which is considered sensitive to precipitation^58^_._ SP ferrimagnets are commonly produced in a relatively wet climate, which allows the conversion of ferric to ferro iron in a reducing environment, whereas hematite is associated with environments having limited precipitation^52^. Therefore, the precipitation signal captured by SP ferrimagnetic minerals is highlighted by normalization to the hematite content^58^. The values of χ_FD_/HIRM remain constant and low in the lower part of the sequence but increase after 31 Ma in the upper half of the sequence, with peak values obtained between ca. 30 and 28 Ma (Fig. 3a), therefore suggesting decreased aridity during the late Oligocene.

The past MAP was predicted using the RF-MAP2.0 model, in which a data set is iteratively divided based on commonly analysed elemental oxide predictors (Al_2_O_3_, CaO, MgO, Na_2_O, K_2_O, SiO_2_, Fe_2_O_3_, TiO_2_, MnO, P_2_O_5_) that maximally reduce the variance in response to MAP^43^. The element oxide compositions of the Ulantatal sediment samples obtained from XRF analyses are presented in Fig. 2c. Briefly, the major elemental oxides show little variation, apart from a period during 30.5–28.5 Ma, when MgO, K_2_O, Al_2_O_3_ and Fe_2_O_3_ exhibit peak values (Fig. 2c). Commonly, arid environments favour the accumulation of base oxides (CaO, MgO, K_2_O and Na_2_O), whereas they are easily mobilized in humid conditions with increased weathering^43,63^. Moreover, highly weathered soils and the prevalence of warm and wet climates drive the residual enrichment of refractory metals (TiO_2_) and Al and Fe oxides (Al_2_O_3,_ Fe_2_O_3_), even though Al_2_O_3_ in addition to SiO_2_, P_2_O_5_ and MnO are seemingly the least sensitive MAP predictors^43^. The predicted MAP derived from the elemental oxides ranges from ca. 330 ± 209 to 550 ± 209 mm, with a mean value of 400 mm. MAP remains mostly unchanged in the lower part of the sequence, but increased variation is observed in the upper half, with the highest predicted values at 30.6–28.9 Ma (Fig. 3b).

### Weathering intensity

**Figure 4:**
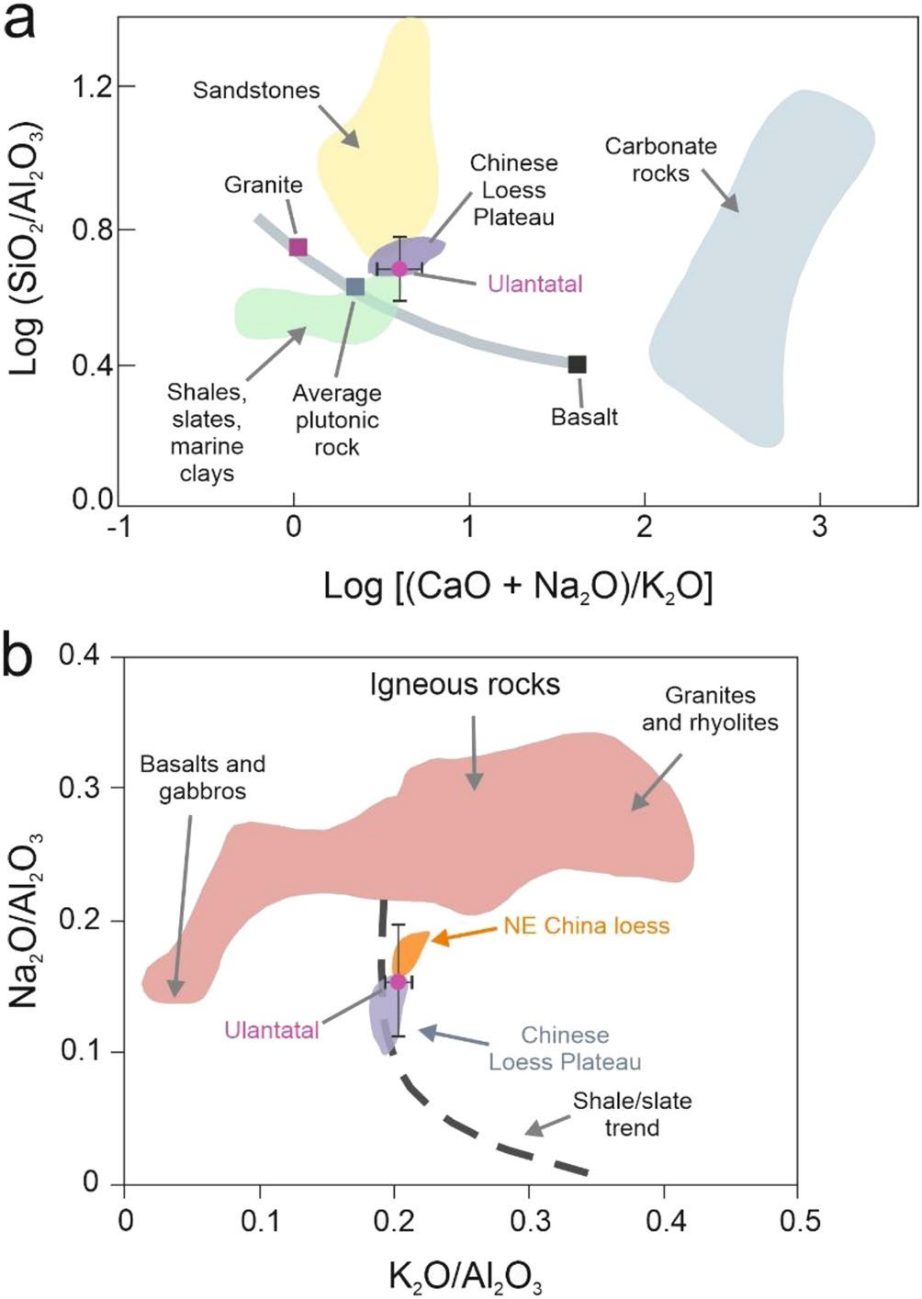
Compositional plots of Garrels and Mackenzie^64^. (**a**) Log [(CaO + Na_2_O)/K_2_O] versus Log (SiO_2_/Al_2_O_3_) depicts the contribution of carbonates plus plagioclase to feldspar on x axis while the y axis is a measure of quartz to feldspars and clay minerals. Ulantatal sediments fall within the field of CLP loess^65–67^, which is expected since aeolian dust has particle size distribution dominated by silt-sized particles. (**b**) utilizes K_2_O/Al_2_O_3_ versus Na_2_O/Al_2_O_3_ as a measure of relative contributions from felsic versus mafic rocks in the x axis while the y axis depicts the relative contributions between igneous rocks and shale. Ulantatal sediments plot mainly to the CLP loess field^68^. The dot represents average composition and the error bars standard deviation.

Based on synchrotron radiation X-ray powder diffraction (SR-XRPD) analyses of Ulantatal bulk sediment samples, the mineral assemblages remain similar across the sequence (Fig. 2b and Supplementary Fig. S3). Quartz (12.8–61.9%, mean = 34.7%), calcite (0.5–39%, mean = 16%) and phyllosilicates are the most abundant minerals, with minor amounts of microcline (0.2–11.3%, mean = 2.4%) and albite (0.6–14.6%, mean = 8%). Of the phyllosilicates, palygorskite (0.4–3.4%, mean = 1.7%) and chlorite (1.8–16.4%, mean = 8.1%) are present along the whole sequence, and the minerals have their maximum values between 30.5 and 28.5 Ma. The bulk chemical composition of the samples mirrors the mineralogy, and it can be studied more in depth with compositional biplots^64^: the average composition of Ulantatal sediments fall within the field of Chinese Loess Plateau (CLP) loess^65–67^ (Fig. 4a). Moreover, this suggests that rocks with an average composition of upper continental crust (approximately granodiorite) were significant suppliers of material to these deposits, as is true for the CLP loess^68^. In Fig. 4b, the composition of Ulantatal sediments plot along the shale trend line mostly within the CLP loess field and distinct from some other Chinese loess. This suggests the sediments have a genetic link to the CLP with variable contributions of sedimentary and metasedimentary rocks, and that the sediments are weathered to different extents as indicated by Na_2_O (plagioclase) depletion.

**Figure 5:**
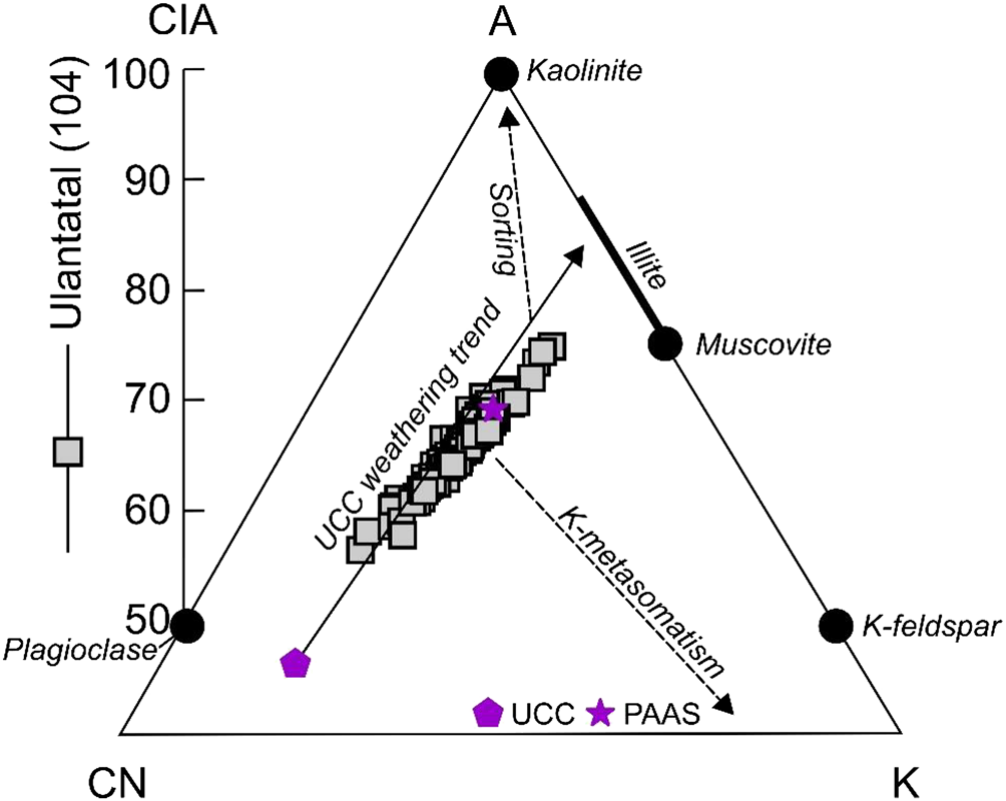
An A–CN–K (Al_2_O_3_–CaO* + Na_2_O–K_2_O) diagram^74^ in which the variably weathered Ulantatal sequence sediments are plotted (grey square symbol). They follow the Upper Continental Crust (UCC, purple polygon symbol) weathering trend close to the composition of Post-Archean average Australian Shale (PAAS, purple star symbol). The range and average value of the chemical index of alteration (CIA) and the number of samples in parentheses are indicated in the left part of the figure. Note that the lower part of the diagram (A < 40) is not shown.

Feldspars are susceptible to increased weathering induced by lowered pH, whereas quartz is recalcitrant in most conditions^58^. Therefore, to understand the weathering characteristics of the Ulantatal sediments, the ratio of (K-feldspar + plagioclase)/quartz (or (Kf + Pl)/Q) was calculated, as it has commonly been used as an indicator of soil weathering, e.g.^69^. Additionally, the silicate weathering intensity and path was estimated from the geochemical data obtained from XRF analyses by calculating three weathering indices, including the well-known chemical index of alteration (CIA, the molar ratio of Al_2_O_3_ to Al_2_O_3_ + CaO* + Na_2_O + K_2_O)^70^, the α_Al_Na^71^ (= (Al/Na)_sample_/(Al/Na)_UCC_, here E is Na), and the weathering index of Yang et al.^72^ especially suited for aeolian sediments (CaO* + Na_2_O + MgO to TiO_2_ ratio). In this study, Ca (CaO*) contents in silicates were corrected from carbonate- and phosphate-bound Ca after^73^. All the three weathering indices show similar behaviour across the sequence, suggesting they are governed by the same mechanism (Supplementary Fig. S5). In concert to Fig. 4b, the (Kf + Pl)/Q ratio indicates that the Ulantatal sediments are weathered to variable degrees (Fig. 3c). This is also corroborated by the CIA values, which range from 58 to 77 (mean = 66) (Fig. 5). The lowest obtained CIA values are slightly above those of unweathered granodiorite and feldspar (45–55), while the highest are similar to muscovite. Transferring these data to an A–CN–K (Al_2_O_3_–CaO* + Na_2_O–K_2_O) diagram^74^ (Fig. 5), all the samples follow the predicted weathering trend from unaltered (Upper Continental Crust, UCC) to values close to the Post-Archean average Australian Shale (PAAS) composition. This single weathering line indicates a parent material with an invariable composition of aluminosilicates^75^. The (Kf + Pl)/Q ratio shows a subtle decrease in silicate weathering from the latest Eocene until ca. 31 Ma (Fig. 3c), after which an evident increase in weathering with larger fluctuations is observed alongside the maximum peak values between 30.2 and 29.5 Ma. After the peak at 29.5 Ma, the weathering intensity shows increased fluctuations until 27 Ma. The lowest weathering intensities were observed at ca. 31 Ma and 28.5 Ma.

Apart from the influence of prevailing climatic conditions on silicate weathering, the depositional rate^76^, provenance^77^ and erosional processes^78^ can all affect pedogenesis. Element concentrations in aeolian dust deposits, and certain weathering indices, are also partially dependent on grain-size changes^72^. Solely inferring the climatic contribution to pedogenesis and weathering is therefore difficult. Our data, however, provides several lines of evidence suggesting chemical weathering in Ulantatal is mainly attributable to climatic factors: (1) bulk sediment chemical composition-based weathering indices do not correlate with grain size (Supplementary Fig. S6), (2) the unidirectional weathering path in the A–CN–K diagram (Fig. 5) suggests a negligible influence of sorting, K-metasomatism, provenance and the tectonic setting on the mineralogical and geochemical characteristics of the studied Ulantatal sediments^75,79^, (3) stability in the properties of antiferromagnetic minerals may indicate no major changes in provenance^37^ (constant L-ratio, Supplementary Fig. S2), and (4) the overall tectonic stability allowed for extensive aeolian dust accumulation in the region^15^. Based on this evidence, it seems likely that the sedimentary and geochemical conditions provide a good opportunity to investigate climate-controlled influences on the Ulantatal proxy records.

### Soil organic carbon

The soil organic carbon (SOC) content varies from ~ 0 to 4.4% (mean = 1.8%). While the SOC record has a lower sampling resolution than the high-resolution χ record, it largely co-varies with χ values throughout the sequence. The SOC increases from the latest Eocene until 28.8–28.4 Ma, where maximum peak values are obtained (Figs. 2c, 3d). The pedogenic origin of SP particles in Ulantatal is corroborated by similar temporal variations in the SOC, since in modern soils and palaeosols, the organic matter content displays a significant positive correlation with the pedogenic magnetic mineral concentration^49,80^. Part of these pedogenic magnetic minerals can be produced by dissimilatory iron-reducing bacteria using organic carbon as an energy source in the reduction of ferric iron to ferro-iron, the excess ferro-iron reacting with ferrihydrite, leading to the production of nanoscale magnetite^81^.

## Discussion and conclusions

### Palaeoclimatic implications

Our results highlight that the general climatic features in the Bayanhot Basin were remarkably stable across the EOT and remained so until around 31 Ma in the Oligocene, after which increased variability in the proxy records is observed. Magnetic susceptibility follows a similar trend to benthic foraminifer oxygen isotope records in the upper part of the sequence, but glacial–interglacial oscillations cannot be distinguished, probably due to the regional climatic overprint (Fig. 2a,d). Furthermore, the dramatic shift in marine δ^18^O records associated with the EOT is not recorded in the Ulantatal proxy records. Instead, the proxy records are strikingly stable at this time, but show fluctuations earlier, ca. 35–34.5 Ma (Fig. 2). Coincident with the changes in magnetic, mineralogical and geochemical characteristics at 34.5 Ma, the depositional setting transitions from a floodplain-dominant environment to aeolian dust lithofacies. Thus, it can be inferred that the lower part of the sequence mainly reflects local sedimentary conditions, whereas after the EOT in the upper part of the sequence, where the χ record follows the benthic oxygen isotope stack to some extent, it probably reflects the regional climatic influence. These stable climatic conditions across the EOT and during the earliest Oligocene are further underlined by the predicted MAP and weathering intensity, which remained unchanged until ca. 31 Ma (Fig. 3b,c). Despite these findings contrast some earlier research done in the nearby regions of the eastern Tibetan Plateau^3,8,12^, the multimillion-year stability in the physical environment, as reflected by the proxy records, is in line with our previous findings on the Ulantatal fossil fauna, which displays a striking lack of change across the EOT and through the early Oligocene^15^. The faunal stability is considered to originate from an environmental change already occurring before the EOT, namely attributed to the long-term and stepwise Eocene aridification.

The late Eocene to early Oligocene semi-arid regime in Ulantatal is also apparent in the predicted MAP values (Fig. 3b), which are within the range commonly recorded in semi-arid regions^43^, and the observed higher hematite abundance in the lower part of the sequence (Fig. 2a), as hematite formation is favoured in prolonged moderate dry (MAP ~ 350–400 mm) conditions, e.g.^38,49,82^. While the southern parts of Alashan region experienced reduced precipitation resulting from the drastic cooling at the EOT^3^, the Northern Alashan arid lands may have been arid for a long time^83^, possibly from the latest Eocene^15^, as suggested also by our new data. Global cooling at the EOT, therefore, may have not abruptly increased aridity in the region during this time, and hence no dramatic shifts in precipitation and weathering intensity can be observed across the critical climatic transition. In line with the muffled response in the Ulantatal proxy records, recent climate model simulations as well as terrestrial proxy data point to a more gradual and smaller drawdown of CO_2_ during the transition than commonly recognized^84,85^. Therefore, the environmental stability in Ulantatal across the EOT might be best explained with the combined effects of locally stable hydrological conditions and a more gradual drawdown of atmospheric CO_2_, both intimately linked with the weathering cycle^86^. After the EOT, the gradual increase in the (Kf + Pl)/Q ratio indicates weakening of silicate weathering during the early Oligocene (Fig. 3c), which probably originates from the continuously cooling and drying terrestrial climate after the Oi-1 glacial, associated with the decline in atmospheric CO_2_ and full Antarctic glaciation^8^. In combination with the sedimentological and faunal characteristics of the Ulantatal sequence^15^, it is apparent that the region has been semi-arid at least since the latest Eocene.

The gradual aridification trend starting from the latest Eocene culminates at 31 Ma, when the lowest weathering intensity is observed. This event can be assigned to the Early Oligocene Aridification Event (EOAE) recorded in Central Asian terrestrial records^10^ and can be linked to the Oi-2 glacial^60^. The termination of the aridification event, and hence the Oi-2 glacial, is followed by warming and an increase in humidity. This change is reflected in the Ulantatal records by a decrease in the (Kf + Pl)/Q ratio, coinciding with lower SOC and higher predicted MAP values, in addition to lower hematite and increased relative magnetite nanoparticle abundance. This is indicated by a higher χ_FD_/HIRM ratio, used as a magnetic humidity (monsoon) proxy^58^ (Fig. 3a–d). Furthermore, the sedimentary features in the upper portion of the sequence display occasional increases in thin laminated clays/clayey silts, some of which contain abundant rip-up clasts, generated by brief flooding events^15^. These are all indicative of the environment changing to a less arid state. Accompanying these changes in the proxies and sedimentary features, the faunal diversity is highest in Ulantatal. Elsewhere, however, the mammal faunas in Central Asia also experienced an optimum phase, called the Late Oligocene Optimum (LGO), which was probably related to reduced aridity stress, enabling the development of ample vegetation that supported herds of large mammals^10^.

This savanna-like environment was warm and probably received most of the rainfall seasonally, as suggested by the flooding events. In addition, rainfall appears to have a greater impact than temperature on pedogenesis and weathering intensity, which control the magnetic enhancement of aeolian dust deposits^35,36,52^. An increase in relative humidity would promote the formation of iron hydroxides at the expense of iron oxides^87^, whereas a higher temperature coupled with increased rainfall would enhance chemical weathering and the enrichment of iron (hydr)oxides through secondary iron oxide production^88^. This is similar to what we infer has transpired in the Ulantatal region based on the concurrent increase in iron oxide and the hematite-normalized nanoscale magnetite content (χ_FD_/HIRM) (Figs. 2c, 3a), and the intensification of silicate weathering (Fig. 3c). If the rainfall dominantly occurred during a wet and warm season, it may have led to a decrease in soil pH coupled to the depletion of dissolved oxygen, which then retarded the oxidation of ultrafine ferrimagnets^52^, while the lowered pH also increased feldspar weathering^89^. If the dry seasons were colder, the oxidation would have still been halted due to reduced bacterial activity^52^, but would also have made the soil environment more alkaline, which is optimal for the formation of the clay mineral palygorskite^90^. Palygorskite peak values between ca. 31 and 29 Ma (Supplementary Fig. S2) are coincident with the observed peak weathering intensity values and increased abundances of Mg, K, Al and Fe oxides (Fig. 2c). This is because increased weathering can typically lead to the retention of Al and Fe, and episodic rainfall in warm environments leads to increased activities of Mg and K, which in turn favours the formation of palygorskite through transformation from other phyllosilicates^91^. At the same time, SOC contents are lower (Fig. 3d) due to the increased weathering and subsequently lowered organic carbon burial^92^.

### Driving mechanisms

Since the peak weathering and rainfall proxy intensities between 30 and 29 Ma are also concurrent with a peak in atmospheric CO_2_ levels^59^ (Fig. 3), it may point to warm greenhouse-like conditions during this relatively brief interval. The climate as the dominant driving mechanism for the observed changes in the proxies is supported by the environmental conditions returning to an arid state at around 28.5 Ma, when the weathering intensity and MAP show a clear decrease associated with a decline in CO_2_ (Fig. 3). Interestingly, this interval is nearly synchronous with the dramatic drop in global sea level^61^ and the mid-Oligocene Glacial Maximum (OGM)^10^, the onset of which corresponds to the Oi-2a glacial (ca. 28 Ma) in marine oxygen isotope records^62^. While the OGM has not previously been evidenced by terrestrial geophysical or geochemical records^10^, it is concurrent with the Mid-Oligocene Reorganization (MOR), during which Asian terrestrial faunas faced a major turnover^10^, also evident in the Ulantatal fauna^93^. Previously, the MOR has been considered to have been triggered by some climatic parameter, such as a change in seasonality and/or temperature^10^. The stability in tectonic and sediment source conditions, as evidenced by the lack of major grain-size variations^15^, a mostly unchanged L-ratio (Supplementary Fig. S3) and the unidirectional weathering path of Ulantatal sediments (Fig. 4), renders secondary alteration and/or changes in provenance an unlikely source for the observed change in the weathering regime to be predominated by physical over chemical weathering and the decrease in rainfall. In addition, the near-synchronicity of these changes with the Oi-2a glacial suggests a temperature drop being the most likely candidate for the observed aridification event, probably driven by the declining atmospheric CO_2_. This arid phase is then again shifted to decreased aridity towards the late Oligocene. Despite the apparent decrease in CO_2_ levels towards the late Oligocene, global sea surface temperature reconstructions based on TEX_86_ indicate a warming trend and a low meridional gradient, similar to the late Eocene setting also persisting after the EOT^6^, ultimately leading to Late Oligocene Warming (LOW). Furthermore, soil carbonate clumped isotope evidence from the nearby Xining Basin suggests gradual warming beginning from 30 Ma, which lasted until around 27 Ma, when the highest temperatures were attained^14^. This could have driven an intensified hydrological cycle, promoting chemical weathering and explaining the predicted higher rainfall and increase in weathering observed around 27 Ma (Fig. 3), before the onset of LOW.

As for the source of the moisture, the unique location of the Ulantatal sequence between the rising Tibetan Plateau and the CLP provides an opportunity to hypothesize two plausible but contrasting moisture transport routes. Firstly, the moisture could have mainly been carried in the winters by the prevailing Westerlies and provided by the close proximity of the highstand proto-Paratethys Sea^28^ during the warm periods. This effect may have been strengthened by the minor uplift event of the Helan Mountains around 30 Ma^45^, which probably altered the orographic characteristics of the region and forced increased rainfall. Alternatively, it is possible that a strengthened East Asian Summer Monsoon (EASM) system could have been the source of moisture. Currently, the Helan Mountains act as the northwestern boundary of the EASM^33^, which has been suggested to have been active at least since the late Oligocene with modern-like strength and seasonality^94^. The possible increase in temperatures during this period and a simultaneous strengthening of summer monsoonal circulation would be expected, since continental summer temperatures govern moist static energy, which in turn controls monsoonal intensity^95^. Therefore, the variability in MAP and weathering intensity in the upper part of the Ulantatal sequence may have originated from the EASM, driven by astronomically forced Antarctic ice sheet oscillations^96^. This is also corroborated by the coupling of aridity events in Ulantatal with the marine oxygen isotope events. However, since the uplift event of the Helan Mountains occurred around 30 Ma, a moisture barrier may have already been in place before the most intensive phase of weathering and rainfall peaks observed in the Ulantatal proxy records, mitigating a possible EASM influence within the Bayanhot Basin and suggesting another route of moisture transport. Hence, if the late Oligocene proto-Paratethys Sea was as extensive as suggested^28^, it still probably had some effect on the land–sea pressure gradient, hampering the monsoonal circulation, and would also have shortened the moisture transport pathway of the Westerlies during the highstands, which instead may have led to a dominantly westerly-derived moisture flux to the region.

To summarize, this study provides an environmental reconstruction of one of the sporadically investigated late Eocene to Oligocene-aged aeolian dust deposits in eastern Central Asia through the first multiproxy record of the Ulantatal sequence, which is situated on the western limit of the Chinese Loess Plateau. Specifically, we show that the strikingly stable semi-arid conditions predominating from the latest Eocene and across the EOT in the region are followed by a shift to decreased aridity and increased climatic oscillations at around 31 Ma, inferred from an increase and variability in the chemical weathering intensity, rainfall, soil organic carbon content and the abundance of hematite-normalized pedogenic nanoscale magnetite, which can be used as a proxy for monsoon intensity^58^. We conclude that the stability in the multiproxy record in the lower part of Ulantatal sequence is probably caused by the tranquil state of the physical environment, driven and maintained by the long-term Eocene aridification of Asian interiors^20^ accompanied with a smaller and a more gradual drawdown of atmospheric CO_2_ across the EOT than commonly realized^84,85^. The aridification trend observed in Ulantatal culminated at 31 Ma, and was likely enhanced by global cooling and the drawdown of atmospheric CO_2_ since the Oi-1 glacial at ~ 33.8–33.6 Ma^8^. On the other hand, we attribute the changes observed after 31 Ma to be primarily caused by the climatic warming associated with the termination of the Oi-2 glacial and the EOAE, possibly enhanced by incursion of the proto-Paratethys Sea^28^, the transient atmospheric CO_2_ peak seen in the marine alkenone record^59^ and the local uplift event of the Helan Mountains^45^, which changed the hydrological and orographic characteristics of the region. However, the role of possible intensification of the East Asian Summer Monsoon system in the observed changes cannot be fully excluded. The regional climatic influence is further underlined by the termination of the warm period, concurrent with the LGO, during which the environment probably changed to Serengeti-like conditions^10^, which transitioned back to increased aridity. This second arid phase in the Ulantatal record can be linked to the OGM and thus the Oi-2a glacial at ca. 28 Ma, which to our knowledge is the first time in eastern Central Asian terrestrial geophysical and geochemical records. Therefore, investigation of more high-resolution terrestrial records in the late Eocene and Oligocene of Central Asia is crucial in formulating a comprehensive understanding of the influence of the dramatic climatic reorganization at the EOT and in its aftermath on terrestrial systems, the evolution of the EASM and the possibly competitive roles of the EASM system and the highstand late Oligocene proto-Paratethys Sea in the moisture dynamics of the region.

## Methods

### Rock magnetism

Magnetic measurements were carried out at the Solid Earth Geophysics Laboratory of the University of Helsinki, Finland. Mass normalized magnetic susceptibility (χ) measurements were undertaken at two operating frequencies of 0.51 kHz (χ_lf_) and 8.1 kHz (χ_hf_) with a field intensity of 320 A/m using a ZH Instruments SM100 Magnetic Susceptibility Meter and software. χ is defined as the ratio between the induced magnetization of a material and the applied magnetic field^37^. When susceptibility increases (decreases), it indicates that the amount of (all) magnetic material increases (decreases), responding to the rate of magnetic influx^97^. The absolute (χ_FD_ = χ_lf_ − χ_hf_) and relative frequency dependency of susceptibility (χ_FD(%)_ = [(χ_lf_ − χ_hf_)/χ_lf_] × 100 (%)) was calculated. χ_FD_ and χ_FD(%)_ are used to detect superparamagnetic (SP) grains from stable single domain (SSD) grains and measure their concentration^37,98^.

Saturation isothermal remanent magnetization (SIRM) of 3 T was imparted using a MMPM10 pulse magnetizer. Magnetization was measured with a 2G (WSGI) SQUID. SIRM was AF demagnetized in steps up to 100 mT (IRM_AF100mT_), in addition to 270 mT (IRM_AF270mT_).

Composition was studied by calculating the L-ratio and S-ratio, and by measuring hard isothermal remanent magnetization (HIRM), thermomagnetic curves and hysteresis properties. The L-ratio was calculated as (IRM_AF270mT_/IRM_AF100mT_) after^53^. HIRM was calculated as (0.5 × (SIRM − IRM_AF270mT_) and the S-ratio as (0.5 × (SIRM − IRM_AF270mT_)/SIRM) after^99^. Thermomagnetic analyses of selected powdered samples were carried out using an Agico KLY-3S-CS3 Kappabridge system. Samples were heated from room temperature to 700 °C and cooled back to room temperature, while the bulk susceptibility was continuously measured. Cureval 8.0.2. software (http://www.agico.com) was used to determine the Curie and Néel temperatures. A Princeton Measurement Corporation Micro-Mag TM 3900 model Vibrating Sample Magnetometer (VSM) was used to measure hysteresis properties for selected powdered samples to determine the domain states of the magnetic carriers. The maximum applied field was 1 T. For each sample, 115 FORCs were recorded with an averaging time of 100 ms. FORC diagrams were analysed using FORCinel v. 3.0 (https://wserv4.esc.cam.ac.uk/nanopaleomag/)^100^.

### Bulk sediment geochemistry and RF-MAP_2.0_

The samples were powdered to a size fraction < 37 µm with a Fritsch Pulverisette 6 planetary ball mill in tungsten carbide vessels for 15 min at 350 rpm. Element geochemistry was quantitatively determined with a PANalytical Axios Max wavelength dispersive XRF spectrometer at the Department of Geosciences and Geography of the University of Helsinki. The samples were prepared as fused beads by mixing 0.6 g of sample powder with 6.0 g of Li_2_B_4_O_7_-LiBO_2_-LiBr flux. Uncertainty for all major elements was ≤ 5% in comparison with the MRG-1 standard. Loss on ignition (LOI) was determined externally at 1000 °C with a LECO TGA701 thermogravimetric analyser from ca. 1 g of sample powder. The element concentrations are expressed as wt% or ppm and were recalculated on a volatile-free basis. The RF-MAP2.0 model^43^ was applied to bulk geochemical samples to estimate palaeoprecipitation. The model is based on recursive partitioning via random forest machine learning calibrated on a BU-SI dataset^101^, and is advocated to be used in settings where palaeoclimate constraints estimate MAP at below 1600 mm^43^. These constraints were deduced based on the presence of carbonate in the sediments, fossil leaf MAP estimates in the nearby Qaidam Basin^102^, predicted values of < 1600 mm based on the RF-MAP1.0 model^43^ and modern rainfall^33^. The RF-MAP code is reposited at http://earth.geology.yale.edu/~ajs/SupplementaryData/2019/Lukens/RF-MAP%20code_revised/ and was run on Rstudio 1.4 (https://rstudio.com/).

### Soil organic carbon content

Finely ground samples were measured with a LECO TGA701 thermogravimetric analyser at 550 °C to calculate LOI, a method commonly used to determine the soil organic carbon (SOC) content for loess–palaeosols^103^. Experimental clay correction and soil organic matter (SOM) to soil organic carbon conversion factors were applied^104,105^: SOC = a_T_ × (LOI_T_ − b_T_ × C), where a_T_ (≈ 0.5500) is the carbon content of SOM, LOI_T_ is the mass loss of SOM at temperature T, b_T_ (≈ 0.0772) is the clay correction factor for structural water loss and C is the clay content. The clay content of the samples was approximated to 30% after the highest clay content in^15^.

### X-ray diffraction

The synchrotron radiation X-ray powder diffraction (SR-XRPD) method was performed on ball-milled samples at the Paul Scherrer Institute, Switzerland, with a Swiss Light Source Materials Science beamline MS-X04SA utilizing an undulator source^106^. X-ray energy was set to 25 keV and the exact wavelength was refined from the NIST SRM 640d standard^107^ utilizing Pawley fitting^108^ on Bruker Topas V6 Rietveld software^109^. The exposures were carried out by Debye–Scherrer geometry utilizing a novel high-throughput sample handling approach based on a piezo-driven Vibrating Sample Holder after^110^, and developed further by Stenman Minerals Ab along with personnel of MS-Beamline at Swiss Light Source. Phase identification was conducted with PANalytical Highscore + 4.9.0 software^111^ utilizing the ICDD PDF-4 Minerals 2020 database^112^ and identified phases were quantified by Rietveld phase analysis.

## Supplementary Information

Supplementary Information.

## Data Availability

The datasets generated during and/or analysed during the current study are available from the corresponding authors on request.

## References

[1] Meng J, McKenna MC (1998). Faunal turnovers of Palaeogene mammals from the Mongolian Plateau. Nature.

[2] Zachos J, Pagani M, Sloan L, Thomas E, Billups K (2001). Trends, Rhythms, and Aberrations in Global Climate 65 Ma to Present. Science.

[3] Dupont-Nivet G (2007). Tibetan plateau aridification linked to global cooling at the Eocene-Oligocene transition. Nature.

[4] Pekar SF, Christie-Blick N, Kominz MA, Miller KG (2002). Calibration between eustatic estimates from backstrapping and oxygen isotopic records for the Oligocene. Geology.

[5] Pälike H (2006). The heartbeat of the Oligocene climate system. Science.

[6] O'Brien CL (2020). The enigma of Oligocene climate and global surface temperature evolution. PNAS.

[7] Wade BS, Pälike H (2004). Oligocene climate dynamics. Paleoceanogr. Paleoclimatol..

[8] Ao H (2020). Orbital climate variability on the northeastern Tibetan Plateau across the Eocene-Oligocene transition. Nat. Commun..

[9] Liu Z (2009). Global cooling during the Eocene-Oligocene climate transition. Science.

[10] Harzhauser M (2016). Stepwise onset of the Icehouse world and its impact on Oligo-Miocene Central Asian mammals. Sci. Rep..

[11] Kraatz BP, Geisler JH (2010). Eocene-Oligocene Transition in Central Asia and its effects on mammalian evolution. Geology.

[12] Sun J (2014). Synchronous turnover of flora, fauna, and climate at the Eocene-Oligocene Boundary in Asia. Sci. Rep..

[13] Sun J, Windley BF (2015). Onset of aridification by 34 Ma acroos the Eocene-Oligocene transition in Central Asia. Geology.

[14] Page M (2019). Synchronous cooling and decline in monsoonal rainfall in northeastern Tibet during the fall into the Oligocene icehouse. Geology.

[15] Wasiljeff J, Kaakinen A, Salminen JM, Zhang Z (2020). Magnetostratigraphic constraints on the fossiliferous Ulantatal sequence in Inner Mongolia, China: Implications for Asian aridification and faunal turnover before the Eocene-Oligocene boundary. Earth Planet. Sci. Lett..

[16] Xiao GQ, Abels HA, Yao ZQ, Dupont-Nivet G, Hilgen FJ (2010). Asian aridification linked to the first step of the Eocene-Oligocene climate Transition (EOT) in obliquity-dominated terrestrial records (Xining Basin, China). Clim. Past.

[17] Botsyun S (2019). Revised paleoaltimetry data show low Tibetan Plateau elevation during the Eocene. Science.

[18] Su T (2019). No high Tibetan Plateau until the Neogene. Sci. Adv..

[19] Jia Y (2020). Cenozoic aridification in Northwest China evidenced by paleovegetationevolution. Palaeogeogr. Palaeoclimatol. Palaeoecol..

[20] Meijer N (2019). Central Asian moisture modulated by proto-Paratethys Sea incursions since the early Eocene. Earth Planet. Sci. Lett..

[21] Kaya MY (2019). Paleogene evolution and demise of the proto-Paratethys Sea in Central Asia (Tarim and Tajik basins): role of intensified tectonic activity at ~ 41 Ma. Basin Res..

[22] Bosboom RE (2014). Aridification in continental Asia after the Middle Eocene Climatic Optimum (MECO). Earth Planet. Sci. Lett..

[23] Caves JK (2015). Role of the westerlies in Central Asia climate over the Cenozoic. Earth Planet. Sci. Lett..

[24] Bougeois L (2018). Asian monsoons and aridification response to Paleogene sea retreat and Neogene westerly shielding indicated by seasonality in Paratethys oysters. Earth Planet. Sci. Lett..

[25] Barbolini N (2020). Cenozoic evolution of the steppe-desert biome in Central Asia. Sci. Adv..

[26] Zhang Z, Wang H, Guo Z, Jiang D (2007). What triggers the transition of palaeoenvironmental patterns in China, the Tibetan Plateau uplift or the Paratethys Sea retreat?. Palaeogeogr. Palaeoclimatol. Palaeoecol..

[27] Pound MJ, Salzmann U (2017). Heterogeneity in global vegetation and terrestrial climate change during the late Eocene to early Oligocene transition. Sci. Rep..

[28] Li Q, Li L, Zhang Y, Guo Z (2020). Oligocene incursion of the Paratethys seawater to the Junggar Basin, NW China: insight from multiple isotopic analysis of carbonate. Sci. Rep..

[29] Meijer, N. *et al.* Identifying eolian dust in the geological record. *Earth-Sci. Rev.* (in Press) (2020).

[30] Zhang Y (2014). Cenozoic record of aeolian sediment accumulation and aridification from Lanzhou, China, driven by Tibetan Plateau uplift and global climate. Global Planet. Change.

[31] Carrapa B (2015). Tectono-climatic implications of Eocene Paratethys regression in the Tajik basin of central Asia. Earth Planet. Sci. Lett..

[32] Zhang Z (2016). Lithostratigraphic context of Oligocene mammalian faunas from Ulantatal, Nei Mongol, China. C. R. Palevol.

[33] Xiao S-C, Ding A-J, Tian Q-Y, Han C, Peng X-M (2019). Site- and species-specific climatic responses of two co-occurring shrubs in the temperate Alxa Desert Plateau, northwest China. Sci. Total Environ..

[34] Guo ZT (2008). A major reorganization of Asian climate by the early Miocene. Clim. Past.

[35] Maher BA, Thompson R (1995). Paleorainfall reconstructions from pedogenic magnetic susceptibility variations in the Chinese Loess and Paleosols. Quat. Res..

[36] Liu Q, Deng C, Torrent J, Zhu R (2007). Review of recent developments in mineral magnetism of the Chinese loess. Quat. Sci. Rev..

[37] Liu Q (2012). Environmental magnetism: Principles and applications. Rev. Geophys..

[38] Maxbauer DP, Feinberg JM, Fox DL (2016). Magnetic mineral assemblages in soils and paleosols as the basis for paleoprecipitation proxies: A review of magnetic methods and challenges. Earth Sci. Rev..

[39] Jia J, Lu H, Wang Y, Xia D (2018). Variations in the iron mineralogy of a loess section in Tajikistan during the Mid-Pleistocene and Late Pleistocene: Implications for the climatic evolution in Central Asia. Geochem. Geophys. Geosyst..

[40] Zhang P, Liu WG (2008). Loess sedimentary organic matter records from the central Chinese Loess Plateau and the implication of C/N. Mar. Geol. Q. Geol..

[41] Lu H (2019). 800-kyr land temperature variations modulated by vegetation changes on Chinese Loess Plateau. Nat. Commun..

[42] Li Y (2020). Moisture evolution in Central Asia since 26 ka: Insights from a Kyrgyz loess section Western Tian Shan. Quat. Sci. Rev..

[43] Lukens WE (2019). Recursive partitioning improves paleosol proxies for rainfall. Am. J. Sci..

[44] Zhao J (2021). Provenance and paleogeography of Carboniferous-Permian strata in the Bayanhot Basin: Constraints from sedimentary records and detrital zircon geochronology. Geosci. Front..

[45] Zhao H (2007). Uplift and evolution of Helan Mountain. Sci. China Ser. D Earth Sci..

[46] Yang X, Dong Y (2018). Mesozoic and Cenozoic multiple deformations in the Helanshan Tectonic Belt Northern China. Gondwana Res..

[47] Gomes Rodrigues H, Marivaux L, Vianey-Liaud M (2012). Expansion of open landscapes in Northern China during the Oligocene induced by dramatic climate changes: Paleoecological evidence. Palaeogeogr. Palaeoclimatol. Palaeoecol..

[48] Hunt, C. P., Moskowitz, B. M. & Banerjee, S. K. Magnetic Properties of Rocks and Minerals in *Rock Physics & Phase Relations: A Handbook of Physical Constants* Vol. 3 (ed Ahrens, T. J.) 189–204 (The American Geophysical Union, 1995).

[49] Maher BA (1998). Magnetic properties of modern soils and Quaternary loessic paleosols: Paleoclimatic implications. Palaeogeogr. Palaeoclimatol. Palaeoecol..

[50] Lovley DR, Stolz JF, Nord GL, Phillips EJP (1987). Anaerobic production of magnetite by a dissimilatory iron-reducing microorganism. Nature.

[51] Maxbauer DP, Feinberg JM, Fox DL, Nater EA (2017). Response of pedogenic magnetite to changing vegetation in soils developed under uniform climate, topography, and parent material. Sci. Rep..

[52] Ahmed IAM, Maher BA (2018). Identification and paleoclimatic significance of magnetite nanoparticles in soils. Proc. Natl. Acad. Sci..

[53] Liu Q, Roberts AP, Torrent J, Horng C-S, Larrasoaña JC (2007). What do the HIRM andS-ratio really measure in environmental magnetism?. Geochem. Geophys. Geosyst..

[54] Bloemendal J, Liu X (2005). Rock magnetism and geochemistry of two plio–pleistocene Chinese loess–palaeosol sequences—Implications for quantitative palaeoprecipitation reconstruction. Palaeogeogr. Palaeoclimatol. Palaeoecol..

[55] Thompson R, Oldfield F (1986). Environmental Magnetism.

[56] Liu Q (2005). Temperature dependence of magnetic susceptibility in an argon environment: Implications for pedogenesis of Chinese loess/palaeosols. Geophys. J. Int..

[57] Özdemir Ö, Dunlop DJ (1996). Thermoremanence and Néel temperature of goethite. Geophys. Res. Lett..

[58] Nie J (2017). Dominant 100,000-year precipitation cyclicity in a late Miocene lake from northeast Tibet. Sci. Adv..

[59] Zhang YG, Pagani M, Liu Z, Bohaty SM, DeConto R (2013). A 40-million-year history of atmospheric CO_2_. Phil. Trans. R. Soc. A.

[60] Miller KG, Wright JD, Fairbanks RG (1991). Unlocking the Ice House: Oligocene-Miocene oxygen isotopes, eustasy, and margin erosion. J. Geophys. Res. Solid Earth.

[61] Miller KG (2020). Cenozoic sea-level and cryospheric evolution from deep-sea geochemical and continental margin records. Sci. Adv..

[62] Hauptvogel DW, Pekar SF, Pincay V (2017). Evidence for a heavily glaciated Antarctica during the late Oligocene “warming” (27.8–24.5 Ma): Stable isotope records from ODP Site 690. Paleoceanography.

[63] Stinchcomb GE (2016). A data-driven spline model designed to predict paleoclimate using paleosol geochemistry. Am. J. Sci..

[64] Garrels RM, Mackenzie FT (1971). Evolution of Sedimentary Rocks.

[65] Gallet S, Jahn B-M, Torii M (1996). Geochemical characterization of the Luochuan loess-paleosol sequence, China, and paleoclimatic implications. Chem. Geol..

[66] Ding ZL, Sun JM, Yang SL, Liu TS (2001). Geochemistry of the Pliocene red clay formation in the Chinese Loess Plateau and implications for its origin, source provenance and paleoclimate change. Geochim. Cosmochim. Acta.

[67] Jahn B-M, Gallet S, Han J (2001). Geochemistry of the Xining, Xifeng and Jixian sections, Loess Plateau of China: Eolian dust provenance and paleosol evolution during the last 140 ka. Chem. Geol..

[68] Muhs DR (2018). The geochemistry of loess: Asian and North American deposits compared. J. Asian Earth Sci..

[69] Yin K (2018). Fe-oxide mineralogy of the Jiujiang red earth sediments and implications for Quaternary climate change, southern China. Sci. Rep..

[70] Nesbitt HW, Young GM (1982). Early Proterozoic climates and plate motions inferred from major element chemistry of lutites. Nature.

[71] Garzanti E, Padoan M, Andò S, Resentini A, Vezzoli G, Lustrino M (2013). Weathering and relative durability of detrital minerals in equatorial climate: Sand petrology and geochemistry in the East African Rift. J. Geol..

[72] Yang S, Ding F, Ding Z (2006). Pleistocene chemical weathering history of Asian arid and semi-arid regions recorded in loess deposits of China and Tajikistan. Geochim. Cosmochim. Acta.

[73] McLennan SM (1993). Weathering and global denudation. J. Geol..

[74] Nesbitt HW, Young GM (1984). Prediction of some weathering trends of plutonic and volcanic rocks based on thermodynamic and kinetic considerations. Geochim. Cosmochim. Acta.

[75] Buggle B, Glaser B, Hambach U, Gerasimenko N, Marković S (2011). An evaluation of geochemical weathering indices in loess–paleosol studies. Quat. Int..

[76] Kukla G (1987). Loess stratigraphy in central China. Quat. Sci. Rev..

[77] Li G, Chen J, Ji J, Yang J, Conway TM (2009). Natural and anthropogenic sources of East Asian dust. Geology.

[78] Lu H, Stevens T, Yi S, Sun X (2006). An erosional hiatus in Chinese loess sequences revealed by closely spaced optical dating. Chin. Sci. Bull..

[79] Bahlburg, H. & Dobrzinski, N. A review of the Chemical Index of Alteration (CIA) and its application to the study of Neoproterozoic glacial deposits and climate transitions in *The Geological Record of Neoproterozoic Glaciations* (eds Arnaud, E., Halverson, G. P. & Shields-Zhou, G.) Ch. 6, 81–92 (Geological Society of London, 2011).

[80] Liu W (2005). Summer monsoon intensity controls C4/C3plant abundance duringthe last 35 ka in the Chinese Loess Plateau: Carbon isotopeevidence from bulk organic matter and individual leaf waxes. Palaeogeogr. Palaeoclimatol. Palaeoecol..

[81] Blundell A, Dearing JA, Boyle JF, Hannam JA (2009). Controlling factors for the spatial variability of soil magnetic susceptibility across England and Wales. Earth-Sci. Rev..

[82] Kämpf N, Schwertmann U (1983). Goethite and hematite in a climosequence in Southern Brazil and their application in classifaction in kaolinitic soils. Geoderma.

[83] Chen Z, Li G (2013). Evolving sources of eolian detritus on the Chinese Loess Plateau since early Miocene: Tectonic and climatic controls. Earth Planet. Sci. Lett..

[84] Hutchinson DK (2021). The Eocene-Oligocene transition: a review of marine and terrestrial proxy data, models and model–data comparisons. Clim. Past.

[85] Lauretano V (2021). Eocene to Oligocene terrestrial Southern Hemisphere cooling caused by declining pCO_2_. Nat. Geosci..

[86] Sheldon ND, Costa E, Cabrera L, Garcés M (2012). Continental climatic and weathering response to the Eocene-Oligocene transition. J. Geol..

[87] Schwertmann U (1971). Transformation of hematite to goethite in soils. Nature.

[88] Kump LR, Brantley SL, Arthur MA (2000). Chemical weathering, atmospheric CO_2_, and climate. Annu. Rev. Earth Planet. Sci..

[89] Nesbitt HW, Fedo CM, Young GM (1997). Quartz and feldspar stability, steady and non-steady-state weathering, and petrogenesis of siliciclastic sands and muds. J. Geol..

[90] Alekseeva TV, Kabanov PB, Zolotareva BN, Alekseev AO, Alekseeva VA (2009). Humic substances of the late Carboniferous palygorskitic paleosol from the southern Moscow Region Russia. Doklady Biol. Sci..

[91] Xie Q (2013). Mechanism of palygorskite formation in the Red Clay Formation on the Chinese Loess Plateau, northwest China. Geoderma.

[92] Petsch, S. T. Weathering of Organic Carbon in *Treatise on Geochemistry* Vol. 12 (eds Holland, H. D. & Turekian, K. K.) 217–238 (Elsevier, 2014).

[93] Angelone C, Zhang Z (2021). Climate change and evolution of early lagomorphs (Mammalia): A study perspective based on new materials of Ordolagus from Nei Mongol (northern China). Vertebrata PalAsiatica.

[94] Vornlocher JR, Lukens WE, Schubert BA, Quan C (2021). Late Oligocene precipitation seasonality in East Asia Based on δ 13 C profiles in fossil wood. Paleoceanogr. Paleoclimatol..

[95] Roe GH (2016). A modeling study of the response of Asian summertime climate to the largest geologic forcings of the past 50 Ma. J. Geophys. Res. Atmos..

[96] Ao H (2021). Eccentricity-paced monsoon variability on the northeastern Tibetan Plateau in the Late Oligocene high CO_2_ world. Sci. Adv..

[97] Evans ME, Heller F (2003). Environmental Magnetism: Principles and Applications of Enviromagnetics.

[98] Liu Q (2005). Quantifying grain size distribution of pedogenic magnetic particles in Chinese loess and its significance for pedogenesis. J. Geophys. Res. Solid Earth.

[99] Bloemendal J, King JW, Hall FR, Doh SJ (1992). Rock magnetism of Late Neogene and Pleistocene deep-sea sediments: Relationship to sediment source, diagenetic processes, and sediment lithology. J. Geophys. Res..

[100] Harrison RJ, Feinberg JM (2008). FORCinel: An improved algorithm for calculating first-order reversal curve distributions using locally weighted regression smoothing. Geochem. Geophys. Geosyst..

[101] Nordt LC, Driese SD (2013). Application of the critical zone concept to the deep-time sedimentary record. Sediment. Rec..

[102] Song B (2020). Qaidam Basin leaf fossils show northeastern Tibet was high, wet and cool in the early Oligocene. Earth Planet. Sci. Lett..

[103] Li N (2020). Quantifying the carbon content of aeolian sediments: Which method should we use?. CATENA.

[104] Hoogsteen MJJ, Lantiga EA, Bakker EJ, Groot CJ, Tittonell PA (2015). Estimating soil organic carbon through loss on ignition: Effects of ignition conditions and structural water loss. Eur. J. Soil Sci..

[105] Hoogsteen MJJ, Lantinga EA, Bakker EJ, Tittonell PA (2018). An evaluation of the loss-on-ignition method for determining the soil organic matter content of calcareous soils. Commun. Soil Sci. Plant Anal..

[106] Willmott PR (2013). The Materials Science beamline upgrade at the Swiss Light Source. J. Synchrotron Radiat..

[107] Black DR (2010). Certification of NIST standard reference material 640d. Powder Diffr..

[108] Pawley G (1981). Unit-cell refinement from powder diffraction scans. J. Appl. Crystallogr..

[109] Coelho AA (2018). TOPAS and TOPAS-Academic: An optimization program integrating computer algebra and crystallographic objects written in C++. J. Appl. Crystallogr..

[110] Sarrazin P, Chipera S, Bish D, Blake D, Vaniman D (2005). Vibrating sample holder for XRD analysis with minimal sample preparation. Adv. X-Ray Anal..

[111] Degen T, Sadki M, Bron E, König U, Nénert G (2014). The HighScore suite. Powder Diffr..

[112] ICDD, P. D. F. *International Centre for Diffraction Data*. (ICDD, 1997).

