# Supplementary material for: Oligocene moisture variations as evidenced by an aeolian dust sequence in Inner Mongolia, China
Source: Sci Rep. 2022 Apr 4;12:5597. doi: 10.1038/s41598-022-09362-y (PMC8980076; doi:10.1038/s41598-022-09362-y)
Supplement: Supplementary file 1 — Supplementary Information. [file 41598_2022_9362_MOESM1_ESM.pdf]

Supplementary File

Oligocene moisture variations as evidenced by an aeolian dust sequence in Inner Mongolia, China

Joonas Wasiljeff[1,2*], Johanna M. Salminen[1,2], Jarkko Stenman[1,3], Zhaoqun Zhang[4, 5, 6], Anu Kaakinen[1*]

[1] Department of Geosciences and Geography, University of Helsinki, P.O. Box 64, Helsinki 00014, Finland

[2] Geological Survey of Finland, P.O. Box 96, Espoo 02151, Finland

[3] Department of Civil Engineering, Aalto University, P.O. Box 11000, Aalto 00076, Finland

[4] Key Laboratory of Vertebrate Evolution and Human Origin of the Chinese Academy of Sciences, Institute of Vertebrate Paleontology and Paleoanthropology, Chinese Academy of Sciences, Beijing 100044, China

[5] CAS Center for Excellence in Life and Paleoenvironment, Beijing, China

[6] University of Chinese Academy of Sciences, Beijing, China

* joonas.wasiljeff@gtk.fi (corresponding author at the Geological Survey of Finland)
* anu.kaakinen@helsinki.fi (corresponding author at the University of Helsinki)

SUPPLEMENTARY FIGURES

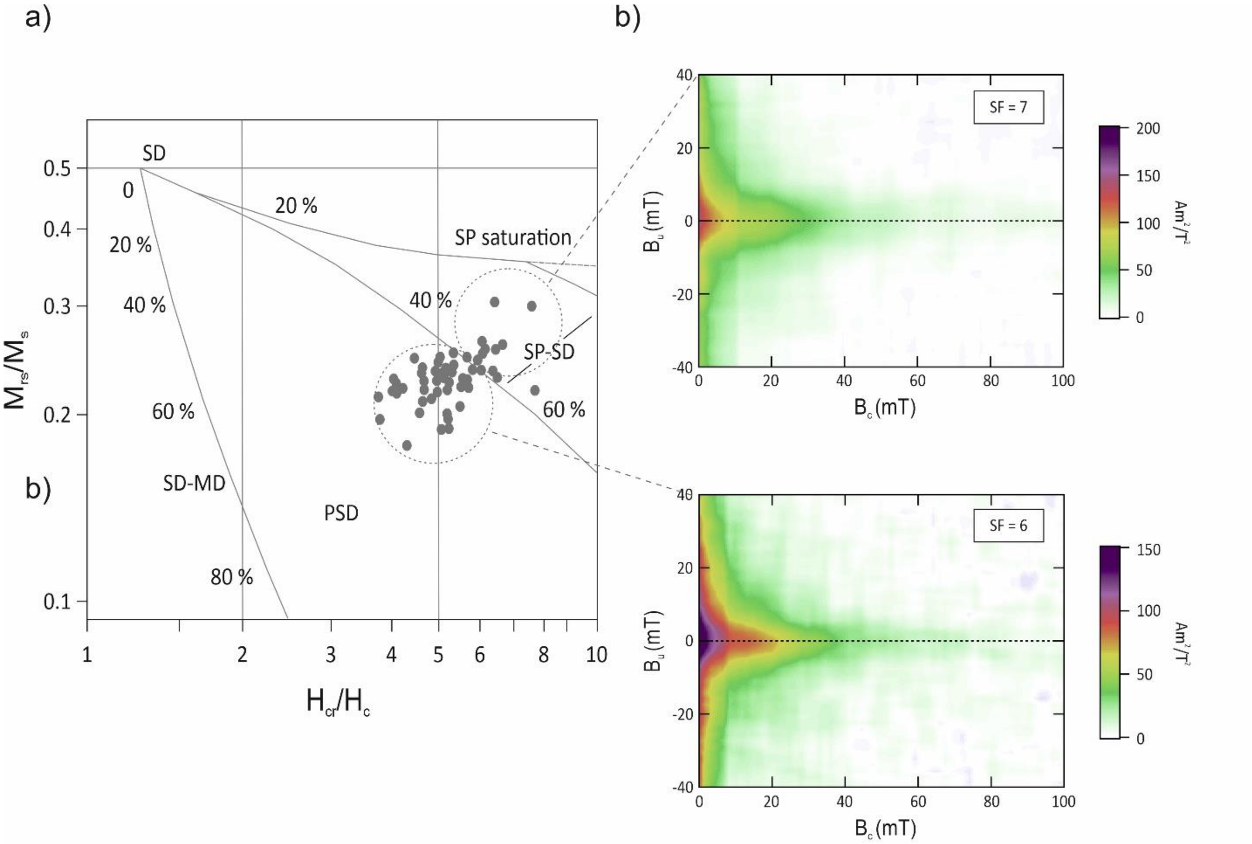

Supplementary Figure S1. a) Day plot[1] with theoretical mixing curves[2] b) first order reversal curve diagrams (FORCs) of representative samples.

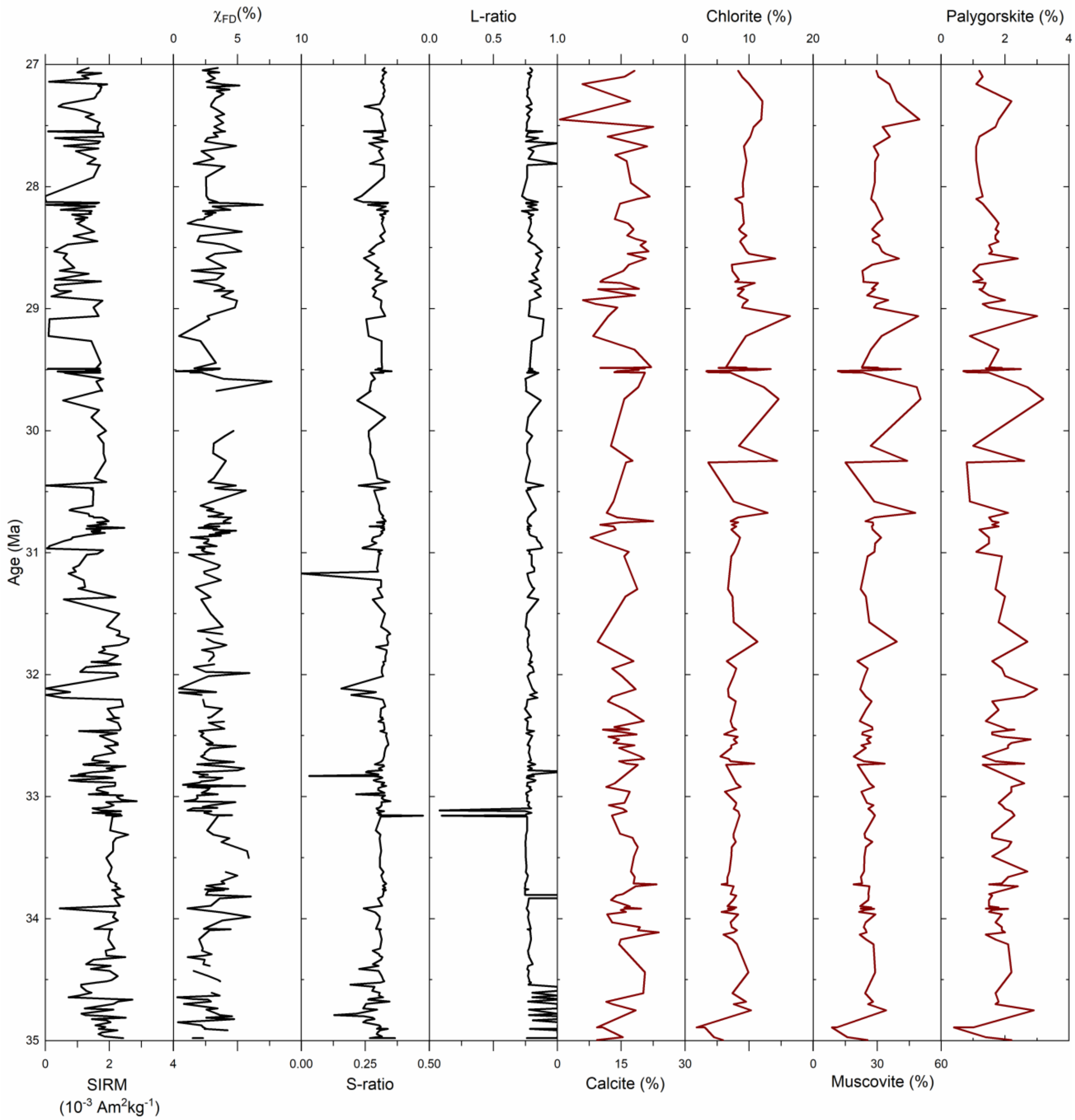

Supplementary Figure S2. Magnetic properties (saturation isothermal remanent magnetization (SIRM), relative frequency dependent magnetic susceptibility χ_{FD}(%), S-ratio, L-ratio) and contents of calcite, chlorite, muscovite, and palygorskite from the Ulantatal sequence.

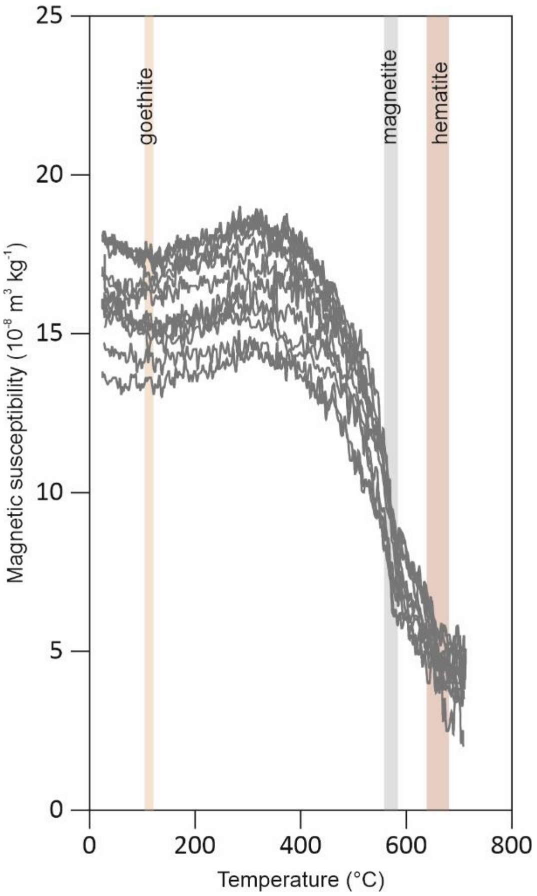

Supplementary Figure S3. Thermomagnetic heating curves of selected samples. Loss of susceptibility between ca. 70 and 130°C indicate the presence of goethite[3], whereas drops near 560-580 °C and ca. 640–680 °C are indicative of the Curie- and Néel-temperatures of magnetite and hematite[4], respectively.

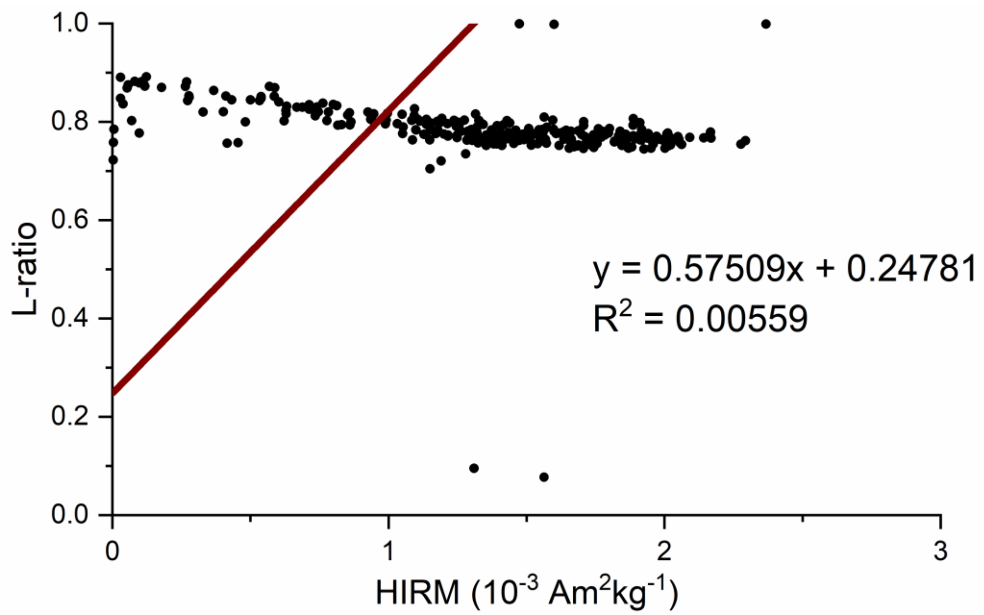

Supplementary Figure S4. Comparison of HIRM and L-ratio from the Ulantatal sequence samples.

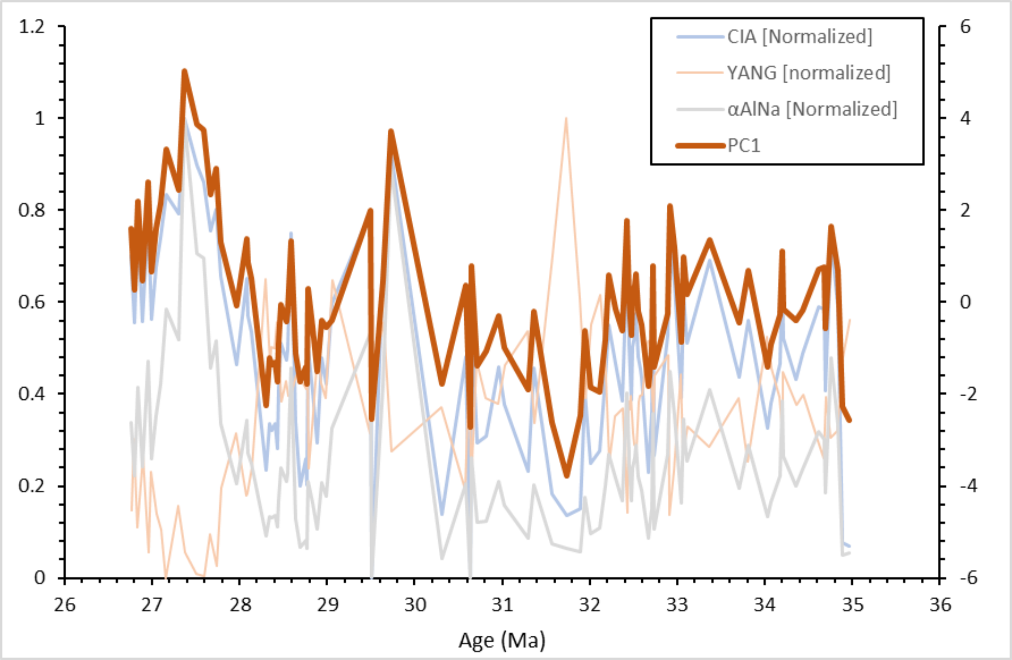

Supplementary Figure S5. Temporal variation of min-max feature scaled weathering indices (CIA, weathering index of Yang et al.[5] (= YANG), $\alpha_{Al}Na$[6]) and principal component 1 (PC1). Correlation principal component analysis (PCA) was performed with OriginPro 2020 software (https://www.originlab.com) to check for consistency of patterns in the normalized chemical weathering indices, and as a result two components could be extracted (PC explains 87% of common variance while PC2 explains 11%). PC1 has strong correlation with the used indices. This suggests that the variations of chemical weathering proxies are predominantly controlled by the same mechanism.

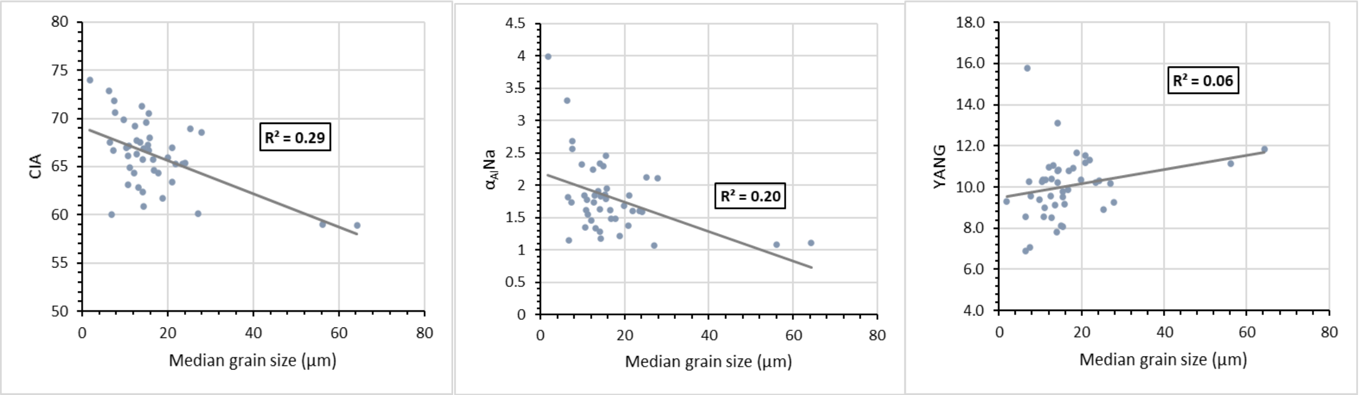

Supplementary Figure S6. Comparison of median grain size of representative Ulantatal sediments and the used chemical weathering indices (CIA, α_{Al}Na, and YANG-index).

REFERENCES

1 Day, R., Fuller, M. & Schmidt, V. A. Hysteresis properties of titanomagnetites: Grain-size and compositional dependence. *Physics of the Earth and Planetary Interiors* **13**, 260 - 267 (1977).

2 Dunlop, D. J. Theory and application of the Day plot (Mrs/Ms versus Hcr/Hc) 1. Theoretical curves and tests using titanomagnetite data. *Journal of Geophysical Research: Solid Earth* **107**, 1 - 22 (2002).

3 Özdemir, Ö. & Dunlop, D. J. Thermoremanence and Néel temperature of goethite. *Geophysical Research Letters* **23**, 921-924, doi:10.1029/96gl00904 (1996).

4 Thompson, R. & Oldfield, F. *Environmental Magnetism*. (Allen & Unwin, 1986).

5 Yang, S. D., Feng; Ding, Zhongli. Pleistocene chemical weathering history of Asian arid and semi-arid regions recorded in loess deposits of China and Tajikistan. *Geochimica et Cosmochimica Acta* **70**, 1695-1709, doi:10.1016/j.gca.2005.12.012 (2006).

6 Garzanti, E. P., Marta; Andò, Sergio; Resentini, Alberto; Vezzoli, Giovanni; Lustrino, Michele. Weathering and Relative Durability of Detrital Minerals in Equatorial Climate: Sand Petrology and Geochemistry in the East African Rift. *The Journal of Geology* **121**, 547-580, doi:10.1086/673259 (2013).
